# MERGE: A Modal Equilibrium Relational Graph Framework for Multi-Modal Knowledge Graph Completion

**DOI:** 10.3390/s24237605

**Published:** 2024-11-28

**Authors:** Yuying Shang, Kun Fu, Zequn Zhang, Li Jin, Zinan Liu, Shensi Wang, Shuchao Li

**Affiliations:** 1 Aerospace Information Research Institute, Chinese Academy of Sciences, Beijing 100094, China; shangyuying21@mails.ucas.ac.cn (Y.S.); kunfuiecas@gmail.com (K.F.); zinanliu_ucas@126.com (Z.L.); wssxm0430@163.com (S.W.); lisc@aircas.ac.cn (S.L.); 2 Key Laboratory of Network Information System Technology (NIST), Aerospace Information Research Institute, Chinese Academy of Sciences, Beijing 100190, China; 3 University of Chinese Academy of Sciences, Beijing 100190, China; 4 School of Electronic, Electrical and Communication Engineering, University of Chinese Academy of Sciences, Beijing 100094, China

**Keywords:** multi-modal knowledge graph, knowledge graph representation, graph attention network, information integration

## Abstract

The multi-modal knowledge graph completion (MMKGC) task aims to automatically mine the missing factual knowledge from the existing multi-modal knowledge graphs (MMKGs), which is crucial in advancing cross-modal learning and reasoning. However, few methods consider the adverse effects caused by different missing modal information in the model learning process. To address the above challenges, we innovatively propose a **M**odal **E**quilibrium **R**elational **G**raph fram**E**work, called **MERGE**. By constructing three modal-specific directed relational graph attention networks, MERGE can implicitly represent missing modal information for entities by aggregating the modal embeddings from neighboring nodes. Subsequently, a fusion approach based on low-rank tensor decomposition is adopted to align multiple modal features in both the explicit structural level and the implicit semantic level, utilizing the structural information inherent in the original knowledge graphs, which enhances the interpretability of the fused features. Furthermore, we introduce a novel interpolation re-ranking strategy to adjust the importance of modalities during inference while preserving the semantic integrity of each modality. The proposed framework has been validated on four publicly available datasets, and the experimental results have demonstrated the effectiveness and robustness of our method in the MMKGC task.

## 1. Introduction

A knowledge graph, such as Wikidata [1], Freebase [2], and WordNet [3], is regarded as a complex semantic network that typically stores vast amounts of factual information in the form of (subjectentity,relation,objectentity) or $\left(e_{s},\;r,\;e_{o}\right)$ for short. In recent years, the emergence of large language models (LLMs) has achieved impressive results in natural language processing (NLP). However, due to limited factual knowledge, LLMs often suffer from hallucinations, generating incorrect factual statements. Given the advantage of knowledge graphs in capturing the intricate representation of entities and their relationships, integrating the knowledge graph with LLMs presents a potentially reasonable solution to overcome these challenges. Nevertheless, due to the difficulty and cost of knowledge acquisition, existing knowledge graphs often contain invalid triplets with missing entities or relations, leading to sparsity and incompleteness. Therefore, to continuously improve the construction of knowledge graphs, researchers have proposed the task of knowledge graph completion (KGC) to promote knowledge discovery.

**Figure 1:**
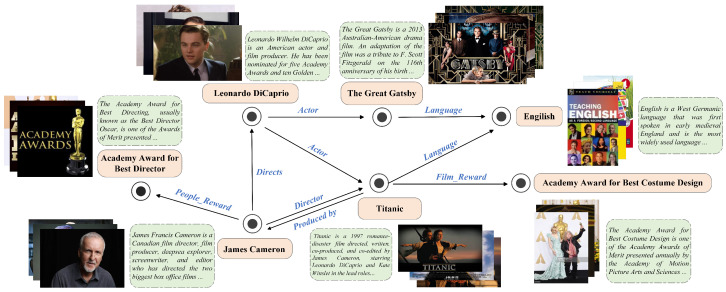
An example of multi-modal knowledge graph in the film domain. The visual information of the entity “Titanic” can be represented by the movie posters and stills, while its textual information can be represented by sentence-level descriptions. Each entity is equipped with text descriptions and multiple images.

Traditional knowledge graph completion methods such as TransE [4] primarily consider the structural characteristics between entities. These methods measure the validity of each triplet through knowledge graph embedding techniques, optimizing scoring functions to obtain entity and relation representations. The real world consists of a variety of modal information [5], where entity description enhances the understanding of abstract concepts, and additional image information visually aids in distinguishing entity attributes and categories. To bridge the information gap in traditional knowledge graphs, researchers have collected and enriched the entity with textual and image information, extending traditional knowledge graphs to multi-modal knowledge graphs. Nowadays, multi-modal knowledge graphs have been widely utilized in various knowledge-driven downstream tasks, such as object detection [6,7], fake detection [8,9], and sentiment analysis [10,11]. In order to address the sparsity in multi-modal knowledge graphs, researchers have proposed the task of multi-modal knowledge graph completion (MMKGC). Figure 1 provides an illustration of the MMKG in the film domain, extracted from the FB15K [12] dataset. Referring to the figure, each entity is accompanied by textual descriptions and multiple images. The entity “James Cameron” and “Titanic” are connected by the relation “Director” and “Produced by”, which can be rewritten as (JamesCameron,Director,Titanic) and (Titanic,Producedby,JamesCameron) for their interrelationship representations.

**Figure 2:**
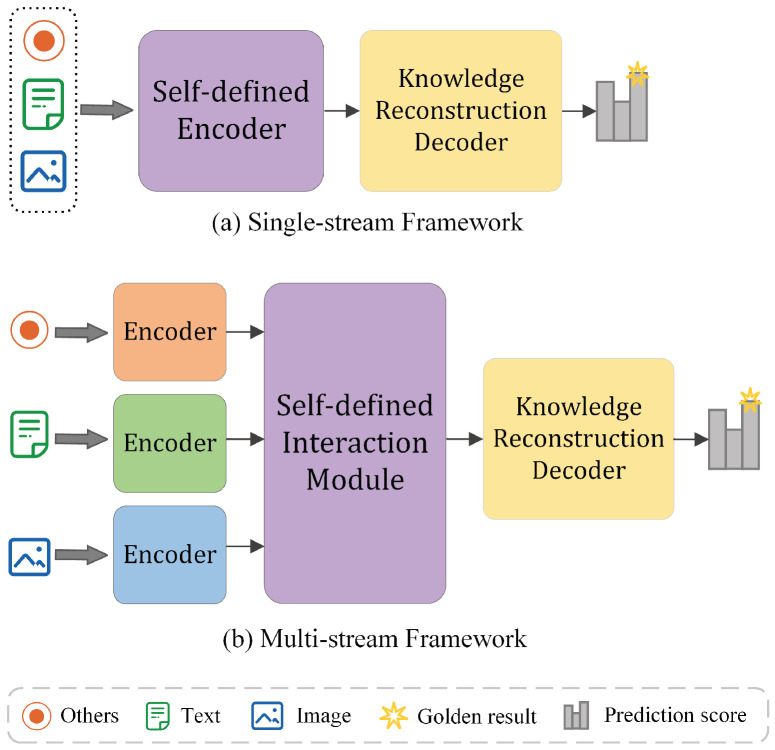
Illustration of the difference between single-stream frameworks and multi-stream frameworks. (**a**) depicts the process of single-stream frameworks, which integrate diverse modal features into an ensemble embedding for the object entity. (**b**) depicts the process of multi-stream frameworks, which encode the information of each modality separately, integrating the multi-modal features through a self-defined interaction module.

In recent years, a series of methods have been proposed for the MMKGC task. Based on the information encoding and fusion approaches, we categorize the methods into single-stream frameworks and multi-stream frameworks. The early MMKGC methods utilize single-stream frameworks, which tightly integrate diverse modal features of an object entity into a single embedding vector before a self-defined encoder. Then, a decoder based on the scoring function of the traditional knowledge representation models was used to reconstruct the encoded features. TransAE [13] uses a five-layer multi-modal encoder to automatically fuse visual and textual features, applying a TransE decoder for entity and relation prediction. We present the illustration of the single-stream frameworks in Figure 2a. Additionally, some researchers have proposed multi-stream frameworks to encode the information of each modality separately and exchange the information through a self-defined interaction module. The process is shown in Figure 2b. IMF [14] proposes a two-stage fusion model, which encodes each modality by a modal-specific encoder, proposing a decision fusion module to better integrate the complementary of different modalities.

**Figure 3:**
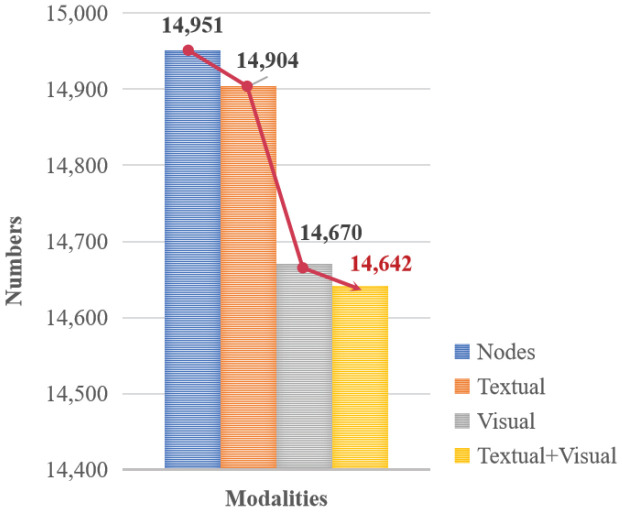
The modality information distribution of 14,951 entities in FB15K dataset.

However, the single-stream frameworks fail to retain the unique characteristics of each modality, and the representational capacity of each modal is affected by the features of other modalities, resulting in great information loss and overfitting issues [15]. On the other hand, multi-stream frameworks have not sufficiently considered the noise impact of the encoded missing modal information during the interaction process, which results in inadequate interpretability of the output entity features. Figure 3 shows the modality information distribution of 14,951 entities in the FB15K dataset. It is obvious to see that visual information is more challenging to collect compared to textual information. Furthermore, the number of entities decreases as the completeness of their assigned modal attributes increases. In fact, there is still a lack of effective discussion on how to effectively address the incomplete multi-modal information issue of entities.

As a heterogeneous graph containing various attributions of nodes and relationships, the multi-modal knowledge graph requires a relation-specific encoding method to achieve precise relational embeddings. Graph neural networks (GNNs) [16] are proposed as a specialized encoding method for graph-structured data to integrate topological structure information and attribute features. By using weighted summation of neighboring nodes to update the hidden states of the object nodes, GNNs can fully exploit the potential connections between nodes and their surrounding objects, which is particularly suitable for obtaining implicit representations of missing modal information in MMKGs. Some researchers have attempted to adopt GNNs into the MMKGC task. Among these efforts, HRGAT [17] stands out. As a single-stream framework, it constructs hyper-nodes with visual, textual, and numerical features, establishing a relational graph attention network to update the representations of hyper-nodes. Nevertheless, HRGAT does not consider the specialized expression among modal features, and thereby, the importance of each modality in representing the object entities has not been adequately quantified.

**Figure 4:**
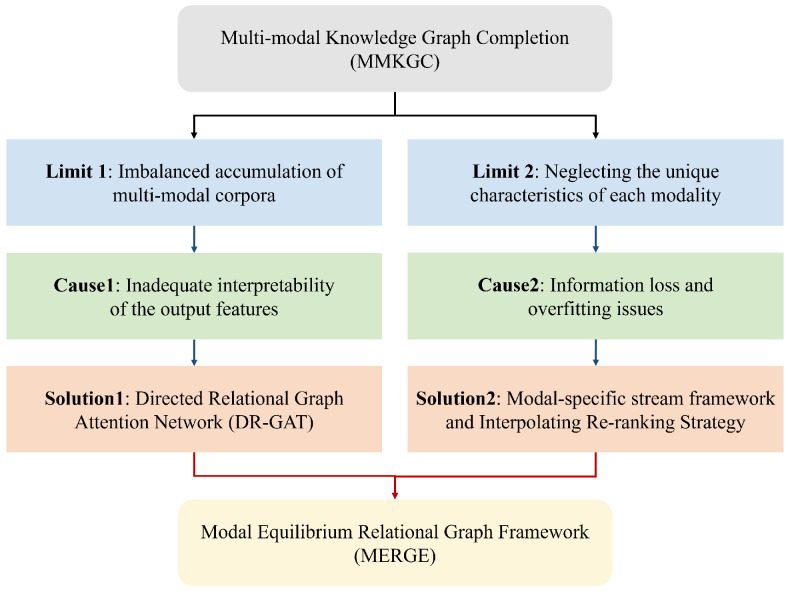
The graphic abstract of the article. The blue and green squares stand for limitations and causes, respectively, while the orange squares stand for the solutions of MERGE.

Inspired by the existing multi-stream frameworks and the graph neural networks, we innovatively propose MERGE, which facilitates multi-modal information interaction while preserving the specific semantic representation of each modality. Figure 4 is the graphical abstract of this paper. To accurately capture the multi-modal information of entities, we first utilize a visual filtering gate to filter the noise images, pre-training the visual and textual features on the CLIP [18] model to obtain semantically aligned modal-specific entity embeddings. Then, we propose three directed relational graph attention networks (DR-GATs) for the structural, visual, and textual modality based on the origin graph structure information of the multi-modal knowledge graphs. By employing the entity–relation combination operations and relation-aware message passing functions, our framework achieves the implicit representation of missing modal features for each entity. The proposed modal-specific DR-GAT can not only align image, text description, and structural information at the entity level but also enhances the interpretability of the entities’ representation embeddings. Subsequently, we adopt decomposition factors in the multi-stream interaction fusion module to capture intra-modal and inter-modal information interaction. Finally, we propose an interpolation re-ranking strategy to reorder the predicted results based on the importance of each modality for the MMKGC task.

We validate the MERGE on FB15K-237-IMG, WN18RR-IMG, DB15K-IMG, and MKG-W datasets, and the results have shown that the proposed framework generally outperforms the baseline models, achieving improvements of 0.5%, 1.9%, 5.8%, and 0.6% in the MRR metric, proving its correctness and robustness. The conducted ablation experiments also demonstrate the effectiveness of each module in our approach. Additionally, we implement a contrast experiment to validate the superiority of the multi-stream framework over the single-stream framework in addressing the MMKGC task based on our proposed DR-GAT network.

In conclusion, we summarize the contributions of this paper as follows:

- We explore a method to address the issue of missing modal information among MMKGs. The experimental results demonstrate that the multi-stream interaction strategy outperforms the single-stream fusion strategy when facing the incomplete multi-modal corpora issue.
- We propose a **M**odal **E**quilibrium **R**elational **G**raph fram**E**work for multi-modal knowledge graph completion, named **MERGE**. This framework achieves modality alignment at both the explicit structural level and the implicit semantic level for structural, textual, and visual features, which preserves the modal-specific information and enhances the interpretability of the represented embeddings.
- Experimental results on four publicly available datasets demonstrate that MERGE outperforms state-of-the-art approaches in the MMKGC task.

## 2. Related Works

Knowledge representation learning essentially converts entities and relationships in a knowledge base into low-dimensional, dense vectors. In this section, we provide an overview of approaches related to our framework.

### 2.1. Knowledge Graph Embedding

Various knowledge graph embedding (KGE) methods have been proposed for the knowledge graph completion (KGC) task, which define scoring functions to project entities and relations into vector space and evaluate the probabilities of valid triplets. We categorize the KGE methods into the following four types:

**Translation-based models**: TransE [4] is regarded as the traditional translation model, known for its simplicity and efficiency, which considers the object entities as the transformation from subject entities via a relation vector. Subsequently, Lin et al. [19] proposed TransR to project entities and relations into different semantic spaces, providing varying weights within different relations. In contrast to the traditional methods that project entity embeddings into the Euclidean space, RotatE [20] defines each object entity as a rotation from the subject entity in the complex space, demonstrating good performance in inferring various relational patterns. Yu et al. [21] proposed TransEllipsoid and TransCuboid to embed each concept as an n-dimensional ellipsoid or cuboid to establish the anisotropy of concept embeddings. Although the translation-based KGE models are popular for their simplicity, they fall short in capturing entities and relationships with complex semantics, which are prone to confusing the semantic representations of similar entities.

**Bilinear models**: The bilinear models represent the relationships between entities by three-dimensional tensors. RESCAL [22] introduces a matrix-based tensor decomposition method that performs representation learning by minimizing the reconstruction error of the predicted triplets. DistMult [23] adopts a bilinear neural network to model multi-relational graphs, simplifying calculations and preventing the overfitting issue of the model. ComplEx [24] extends the representation space to the complex space, showing advantages in modeling asymmetric relationships. These approaches demonstrate superiority in expressing complex relationships; however, the operations on high-dimensional tensors reduce the training and inference efficiency.

**Neural network-based models**: The emergence of deep neural networks has facilitated the utilization of convolutional neural networks (CNNs) in knowledge graph representation. ConvE [25] first leverages convolutional and fully connected layers to facilitate interactions between entities and corresponding relations. CrossE [26] introduces an interaction matrix to model the crossover interactions between entities. InteractE [27] employs feature permutation, checkered reshaping, and circular convolution to capture diverse heterogeneous interactions, enhancing the model’s expression ability. The deep neural networks have demonstrated superior performance on some knowledge graph datasets due to their effectiveness in parameterization. However, these approaches do not fully utilize the structural features of knowledge graphs and their output embeddings lack interpretability.

**GNN-based models**: Traditional deep learning methods have limited performance when dealing with non-Euclidean data. Therefore, the graph neural networks have been proposed to aggregate entity features for the KGC task by leveraging the structural information of knowledge graphs. GCN [28] first proposes the graph convolution method to aggregate information from neighboring entities. R-GCN [29] associates the weight matrix with different types of relations to account for the influence of relationships between entities. However, the R-GCN method only establishes a relation embedding matrix without incorporating the relation embeddings into the update process. Then, CompGCN [30] proposes to utilize an entity–relation composition operation to learn the joint embeddings of entities and relations. SR-GNN [31] develops two semantic aggregation modules to pay attention to the influence of semantic information among neighboring entities.

Nevertheless, with the continuous enrichment of knowledge graph information, the aforementioned KGE methods are no longer sufficient to deal with the growing complexity of the data demands. These approaches primarily focus on handling structural fact triplets, neglecting the potential value of multi-modal information.

### 2.2. Multi-Modal Knowledge Graph Representation

The mainstream methods for MMKGC tasks typically extend traditional KGE models with diverse modal features. Based on different multi-modal information interaction strategies, we classify the multi-modal knowledge graph representation methods into single-stream frameworks and multi-stream frameworks:

**Single-stream frameworks**: These methods typically integrate various modal features of object entities into a single vector, considering the semantic space of the fused embeddings as the representation space. IKRL [32] is the first to introduce the image embeddings into the TransE scoring function, utilizing the visual information of entities. TransAE [13] further integrates image and description features of entities, extending TransE into a multi-modal scoring function to decode the predicted triplets. Subsequently, KBLRN [33] considers expanding the available modal information by introducing numerical features for entity representations. However, substantial noise is contained in image copra in MMKGs, which may interfere with the effectiveness of the information fusion. To address this, RSME [34] proposes a filter gate and a forget gate to obtain valuable visual embeddings, modeling entity relationships through the ComplEx scoring function. Considering the limited performance of translation-based models in handling complex relationships, KMAGCN [35] adopts the graph neural network to model the structural information of the knowledge graph, integrating textual information, visual information, and knowledge concepts into a unified framework to capture semantic representations of entities. HRGAT [17] further incorporates numerical features into a graph attention network, considering the varying importance of neighboring nodes. CMGNN [36] aggregates information from local neighboring entities under a graph neural network framework with a contrastive learning mechanism. Although the single-stream frameworks have achieved superior performance on the MMKGC task, few consider the specialized expression among modal features, resulting in information loss and model overfitting issues.

**Multi-stream frameworks**: These methods define different modal encoders to first learn the unique features of each modality, and then leverages customized information propagation modules for the interaction of multi-modal features. OTKGE [37] models the multi-modal fusion process as a transportation process that moves different modal embeddings into a unified space, considering the optimal transport strategy for three modalities. However, this method primarily focuses on binary relations, whereas real-world multi-modal information often manifests as beyond-binary relations. IMF [14] utilizes the independent representation of different modalities and applies the contrastive learning method as an additional constraint, providing learnable weight parameters for all modal predictions in the decoding module to better integrate the complementary of different modalities. However, the contextual information for each triplet is represented solely through a bilinear outer product between entity and relation embeddings. Both of the aforementioned approaches oversimplify relationships in the real world, failing to account for the semantic associations between neighboring entities and the target entity. In fact, incorporating relation embeddings into the GCN formulation enables more sophisticated modeling of real-world relationships. MMRNS [38] introduces a knowledge-guided cross-modal attention module, constructing a contrastive semantic sampler to enhance the semantic representation of multi-modal entities. Since this method tightly couples textual and visual features together, its scalability is limited. Therefore, the absence of features from one modality can result in a significant degradation in the method’s performance.

Nevertheless, modality completeness cannot always be guaranteed in real-world scenarios. Some researchers have considered introducing generative models such as GAN [39] and VAE [40] to assist in completing modality information or generating labeled data. Zhang et al. [41] proposed MACO to generate missing visual features, designing cross-modal contrastive loss to improve the quality of the generated features. Ref. [42] introduced a weighted semi-supervised DNN-based approach and a GAN-based method to address challenges of limited labeled data and missing unlabeled data in indoor localization. However, assigning values to missing values through specific data may introduce additional noise during the fusion process. Some other methods have considered joint learning approaches. Han et al. [43] proposed a joint training model consisting of two modality-specific encoders and a shared classifier, which implicitly integrates information from different modalities for emotion recognition. From our perspective, such implicit fusion methods are less effective at providing clear insights into the contributions of each modality to the final decision. In contrast, decision-level fusion has been shown to offer greater interpretability and flexibility by combining modality-specific outputs explicitly [44,45].

Therefore, we propose MERGE, a multi-stream framework-based approach, which applies three modal-specific directed relational graph attention networks to fully retain the unique features of each modality, while modeling the complex relationships through the graphical information of knowledge graphs. Moreover, we propose an interpolation re-ranking strategy, leveraging the decision-level fusion of modality-specific decoding scores to mitigate the impact of missing modality data on model performance. Table 1 presents the comparison between MERGE and other existing knowledge graph representation methods.

## 3. The Proposed Method

### 3.1. Preliminary

In this section, we introduce the fundamental concepts utilized in this article and present the definition of the MMKGC task. The knowledge graph can be defined as G={E,R,T}, where E,R, and T denote the set of entities, relations, and known triplets, respectively. Each entity E has three types of information in a multi-modal knowledge graph, including structural information $E_{s}$, textual information $E_{t}$, and visual information $E_{v}$. Triplet T={(s,r,o)} in an MMKG can be interpreted as the connection between the subject entity *s* and object entity *o* via the relation *r*. Following the previous works [30,46], we add the inverse relations $R_{inv}$ = $\left\{r^{-1}|r\in R\right\}$ to the relation set to enable bidirectional information transmission in the knowledge graph. The set of entities, relations, and triplets of an incomplete multi-modal knowledge graph $G^{\prime }=\left\{E^{\prime },R^{\prime },T^{\prime }\right\}$ can be defined as:

(1) $$\begin{array}{c} \begin{array}{c} E^{\prime }=E_{s}\cup E_{t}\cup E_{v} \end{array} \end{array}$$

(2) $$\begin{array}{c} \begin{array}{c} R^{\prime }=R\cup R_{inv}\cup \left\{⊤\right\} \end{array} \end{array}$$

(3) $$\begin{array}{c} \begin{array}{c} T^{\prime }=T\cup \left\{\left(o,r^{-1},s\right)|\left(s,r,o\right)\in T\right\}\cup \left\{\left(s,⊤,s\right)|s\in E\right\} \end{array} \end{array}$$

where ⊤ is regarded as the self-loop relations, ensuring that the information of entity nodes themselves will not be lost during feature updates.

The multi-modal knowledge graph completion task aims to predict the missing head or tail entities in triplets based on the known multi-modal entity set $E^{\prime }$ and relation set $R^{\prime }$, which can be defined as:

(4) $$\begin{array}{c} \begin{array}{c} T=\{\left(s,r,o\right)|s,o\in E^{\prime },r\in R^{\prime },\left(s,r,o\right)\notin T^{\prime }\} \end{array} \end{array}$$

**Figure 5:**
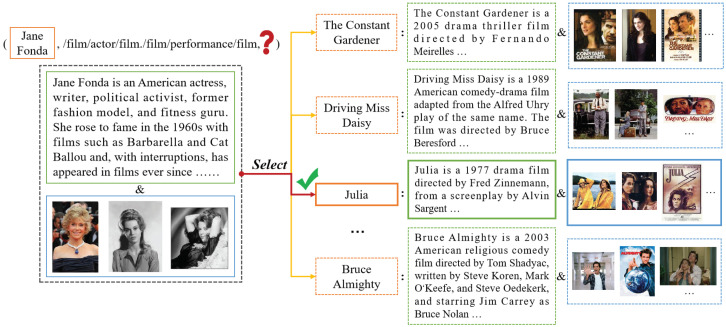
The illustration of the example of tail entity prediction in the MMKGC task, which can be formed as (h,r,?).

To simplify the expression of the MMKGC task, we divide it into the forms of the head entity prediction (?,r,t) and tail entity prediction (h,r,?). Figure 5 illustrates an example of predicting the tail entity in a triplet (JaneFonda,filmactor,?). By taking into account the multi-modal information of all entities and relations in the MMKG, the model will select the entity of the film named Julia that matches the given relationship to accomplish the incomplete triplet.

### 3.2. Framework

**Figure 6:**
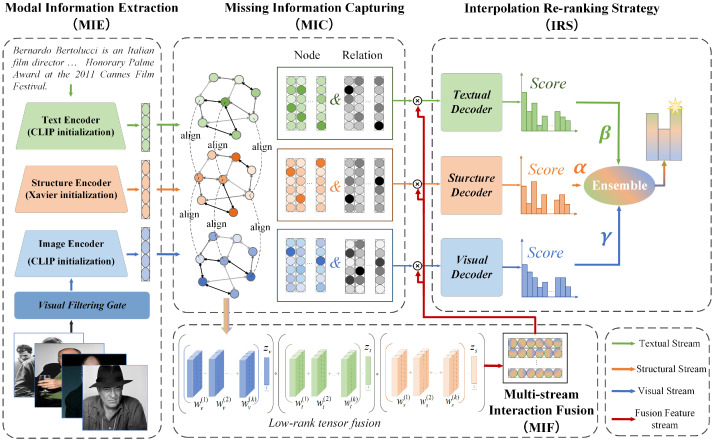
The framework of MERGE for multi-modal knowledge graph completion.

The proposed MERGE adopts an multi-stream encoder–decoder architecture. The overall architecture of MERGE is depicted in Figure 6, which includes four components: modal information extraction, missing information capturing, multi-stream interaction fusion, and interpolation re-ranking strategy. Our framework first utilizes the modal information extraction module to filter and obtain aligned textual and visual features of entities. Then, by defining a directed relational graph attention network for each modality, it produces the implicit representation of the entities’ missing modal features, aligning the structural, textual, and visual features at the entity level. Subsequently, the MERGE employs a low-rank modal fusion method to facilitate intra-modal and inter-modal information interaction. Finally, we propose an interpolation re-ranking strategy to reorder the prediction results based on the importance of each modality while preserving the unique semantics of each modality. The specific functions and processing steps of each part will be explained in detail in the following sections.

#### 3.2.1. Modal Information Extraction

The existing MMKGs consist of entities with various modalities; each represents a different scenario. Therefore, it is crucial to effectively capture information from each modality and address the semantic alignment of the entities’ image–text information.

As a textual-visual pre-trained model, CLIP [18] has demonstrated promising zero-shot performance on various image classification datasets. Through the contrastive learning strategy, CLIP generates representations by maximizing the cosine similarity between the original text and its corresponding image. Thus, to obtain the matching visual and textual features of entities, we first pre-train the image–text pairs of entities based on CLIP. As the Vision Transformer (ViT) [47] can capture more global features than the traditional convolutional neural networks, we adopt the CLIP architecture based on ViT to extract multi-modal features of entities. To be more specific, each entity is equipped with *n* text descriptions $D=\{d_{1},d_{2},d_{3},\ldots ,d_{n}\}$ and *m* corresponding images $I=\{i_{1},i_{2},i_{3},\ldots ,i_{m}\}$. We feed the images into the visual side and the description sequences into the textual side of CLIP, saving the pre-trained model parameters by optimizing the contrastive loss function for further feature extraction.

**Textual Feature Extraction**: To extract entities’ textual description features, we apply the pre-trained textual side of CLIP as our sentence-level feature encoder. The text encoder employs a 12-layer Transformer block of 512 width with eight multi-head self-attention units. Raw texts are transformed into tokens by byte pair encoding [48] with a vocabulary size of 49,152. The input sequence length is limited to 128 and a special [CLS] token is added before as a global aggregation token. The resulting 512-dimensional embedding of the [CLS] token is served as the textual embedding of the entity.

**Visual Feature Extraction**: The image corpora of a practical MMKG contains a large amount of noise images due to the high cost and difficulty of acquisition, which may mislead the model to perform an incorrect inference in the MMKGC task. The noise mainly originates from two sources: incorrect images crawled from the internet and images with limited visual semantic similarity to the related entity. According to the above reasons, it is a challenging problem to filter and choose a typical visual representation of the entity.

**Figure 7:**
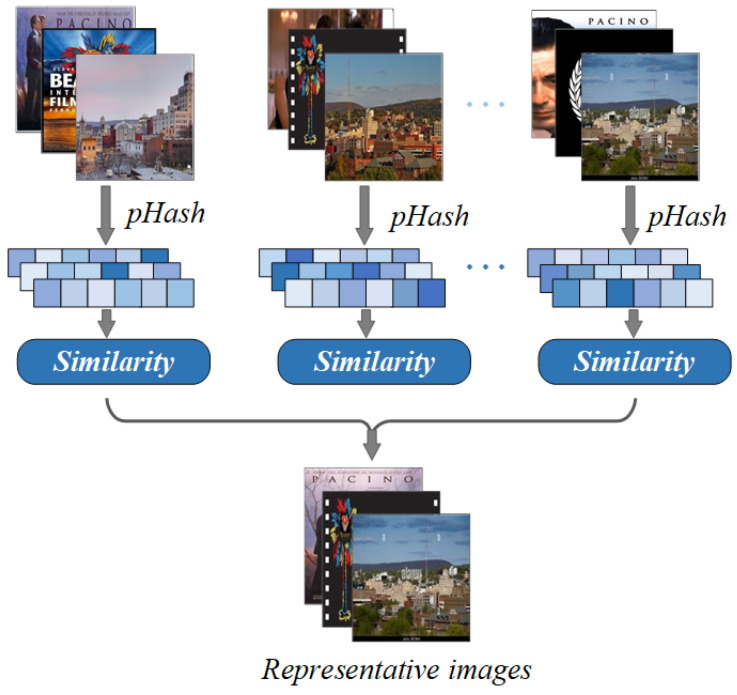
The illustration of the visual filtering gate, which is proposed to select the typical representative image of each entity.

Inspired by the method of [34], we introduce a visual filtering gate before the image encoder to eliminate noisy images from the datasets. Figure 7 is an illustration of the visual filtering gate. Specifically, based on the perceptual hash algorithm (pHash) [49], we first convert the color image to grayscale and resize it to a fixed dimension of 32 × 32. Then, a Discrete Cosine Transform (DCT) [50] is applied to extract the primary features of the image. From the DCT results, we select the top-left 8 × 8 submatrix and generate a 64-bit perceptual hash value based on the average value of this region. Image similarity is then assessed by calculating the Hamming distance [51] between hash values, with a smaller Hamming distance indicating greater similarity.

For a given entity *e*, we can define its image corpora as $I_{e}=\left\{img_{1},img_{2},img_{3},\ldots ,img_{m}\right\}$. The proposed visual filtering gate utilizes the pHash value of the images to identify the typical representative image $img_{e}$ of the entity by similarity calculating function S(·):

(5) $$\begin{array}{c} \begin{array}{c} img_{e}=\underset{img_{s}\in I_{e}}{\arg\max}∥\sum_{i=1}^{m}S\left(p\left(img_{s}\right),p\left(img_{i}\right)\right)∥ \end{array} \end{array}$$

The CLIP visual encoder is established based on “ViT-B/32”, which consists of 12 Transformer blocks and 12 multi-head self-attention units. Images are first converted into patches and a position encoding is added with the encoded image tokens. We also add a [CLS] token at the beginning of the input tokens. After passing through a global pooling function, the 512-dimensional vector of [CLS] is achieved as the visual embedding of the entity.

**Structural Feature Extraction**: To better preserve the graphical structure information in MMKGs, we regard each entity as a graph node and the relations between entities as edges. The Xavier initialization method is employed to initialize the node and edge features with $d_{0}$ dimensions.

After obtaining the semantically aligned image–text entity features, we project the textual and visual embeddings into the same semantic space as the structural embeddings by trainable weights $W_{t}\in R^{d_{t}\times d_{0}}$ and $W_{v}\in R^{d_{v}\times d_{0}}$, respectively. Here, $d_{t}$ and $d_{v}$ are the dimensions of the extracted textual and visual embeddings. The missing modal features in entities will be initialized by zero vectors. We adopt the modal-specific directed relational graph attention network mentioned in Section 3.2.2 to update the implicit representation of the entities’ missing modal information.

#### 3.2.2. Missing Information Capturing

To retain the unique semantic information of each modality, we adopt a multi-stream framework that constructs a directed relational graph attention network (DR-GAT) for each modality. Algorithm 1 presents the pseudocode of the DR-GAT process. Section 4.5.7 qualitatively analyzes the effectiveness of the multi-stream framework compared with the single-stream framework. The construction of DR-GAT will be introduced in detail in the following content.

According to the graph neural networks, entities are treated as nodes, and relations are regarded as edges. To update the implicit representations of entities in multi-modal knowledge graphs, we employ a message passing function that aggregates information from neighboring nodes through edges. Unlike the traditional GCN-based [28,52] methods, our model utilizes an entity–relation combination operation in the message passing function for information aggregation. Additionally, we employ a relation-aware attention mechanism to capture the importance of neighbors through relational correlation patterns.
**Algorithm 1** The aggregation process of DR-GAT**Input:** Modal-specific embeddings in triplet (s,r,o)**Output:** Entity and relation embedding **e** and **r**  1:**for** L∈(1,2,…,k−1) **do**                                 ▹The layer number of DR-GAT is **L**  2:    $\phi \left(e_{s},e_{r}\right)\leftarrow W_{r}\circ e_{s}+W_{r}\circ e_{s}\circ e_{r}$  3:    $m\left(s,r,o\right)\leftarrow W_{dir}\phi \left(e_{s},e_{r}\right)$  4:    **for** j∈N(s) **do**                                      ▹N(s) is the neighboring set of entity **s**  5:        **for** $h_{a}\in \left(1,2,\ldots ,H\right)$ **do**                                  ▹*H* is the number of attention head  6:           $att_{j}\leftarrow \sigma \left(W_{att}m\left(e_{s},e_{r},e_{j}\right)\right),\;j\in N\left(s\right)$  7:           $\alpha_{j}\leftarrow \mathrm{softmax}\left(att_{j}\right)$                           ▹$\alpha_{j}$ is the attention coefficient of node *j*  8:        **end for**  9:        $e_{o_{j}}\leftarrow \left(\frac{1}{H}\sum_{h=1}^{H}\alpha_{j}^{h}m^{h}\left(e_{s},e_{r},e_{j}\right)\right)$10:    **end for**11:    ${e_{o}}^{k}\leftarrow \sigma \left(\sum_{j\in N(s)}{e_{o}}_{j}^{k-1}\right)$12:    ${e_{r}}^{k}\leftarrow W_{r}^{k-1}e_{r}^{k-1}$13:**end for**14:**return e**, **r**

**Entity–Relation Combining Operation**: Graph neural networks aggregate information from neighboring nodes to the object node through relation edges. Studies in [30] have shown that relations can provide relevant information for entity representation during the information aggregation process. Therefore, we introduce an entity–relation combination operation, which can be defined as follows:

(6) $$\begin{array}{c} \begin{array}{c} e_{o}=\phi \left(e_{s},e_{r}\right) \end{array} \end{array}$$

where ϕ is denoted as the fusion operator, and $e_{s},e_{r},e_{o}\in R^{d_{0}}$ is the corresponding embedding of the subject entity, relation, and object entity. Since the DR-GAT is constructed for each modality, *e* is represent as the textual, visual, and structural embeddings. Inspired by TransE [4], DistMult [23], HolE [53], and CrossE [26], we propose four entity–relation combining operations for the combination of entity and relation information. The origin definitions of their score functions are shown in Table 2. Each entity–relation combining operation is defined as follows:

• **Subtraction (Sub)**: $\phi \left(e_{s},e_{r}\right)=e_{s}-e_{r}$;
• **Multiplication (Mult)**: $\phi \left(e_{s},e_{r}\right)=e_{s}\circ e_{r}$;
• **Circular-correlation (Corr)**: $\phi \left(e_{s},e_{r}\right)=e_{s}★e_{r}$;
• **Crossover interaction (Cross)**: $\phi \left(e_{s},e_{r}\right)=W_{r}\circ e_{s}+W_{r}\circ e_{s}\circ e_{r}$.

Symbol ∘ denotes the Hadamard product, and ★ denotes circular convolution. $W_{r}\in R^{d_{r}\times d_{0}}$ is regarded as a relation-specific matrix, learning interactions between the object node and its corresponding relations. $d_{r}$ is the total number of relations.

**Figure 8:**
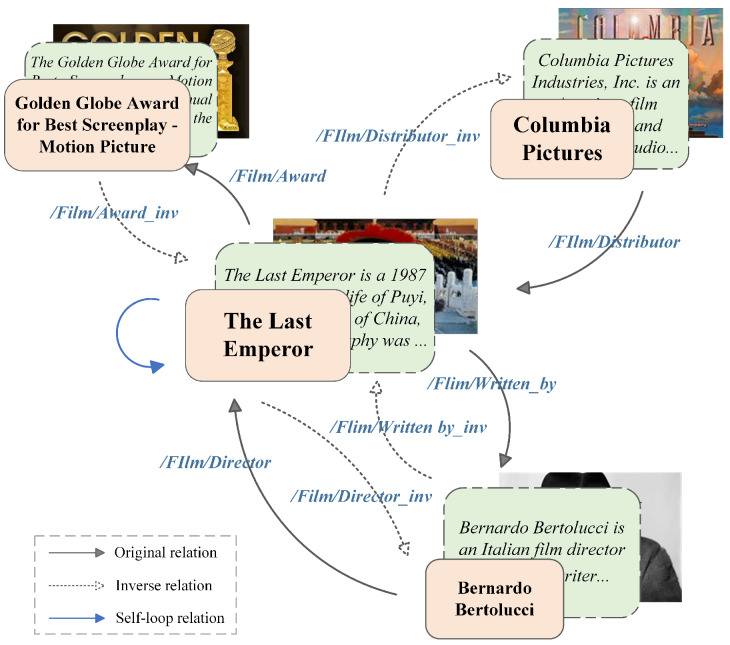
An example of a directed relational multi-modal knowledge subgraph. Each entity is equipped with three modalities and is connected by relational edges to form a directed graph.

**Relation-aware attention mechanism**: Referring to Equation (4), we define the MMKG as a graph with three types of edges: original, inverse, and self-loop. In order to represent each type of edge, we propose the direction-specific weights. Figure 8 depicts an example of a directed relational multi-modal knowledge subgraph. The solid arrows denote original relations, while dotted arrows denote inverse relations. The message passing function can be defined as follows:

(7) $$\begin{array}{c} \begin{array}{c} m\left(s,r,o\right)=W_{dir}\phi \left(e_{s},e_{r}\right) \end{array} \end{array}$$

(8) $$\begin{array}{c} \begin{array}{c} W_{dir}=\left\{\begin{array}{cc} W_{O}, & r\in R\\ W_{I}, & r\in R_{inv}\\ W_{S}, & r\in \{⊤\} \end{array}\right. \end{array} \end{array}$$

$W_{dir}\in R^{d_{1}\times d_{0}}$ is a parameter specific to relation types that is defined by Equation (8). Here, O,I, and *S* represent original, inverse, and self-loop directions. $d_{0}$ and $d_{1}$ represent the input and output dimensions. To evaluate the varying importance of neighboring nodes during information aggregation, we adopt a single-layer dense projection to calculate the relation-aware attention of the adjacent neighboring set N(o) of the central node *o*:

(9) $$\begin{array}{c} \begin{array}{c} att_{j}=\sigma \left(W_{att}m\left(j,r,o\right)\right),\;j\in N\left(o\right) \end{array} \end{array}$$

We parameterize the projection matrix by $W_{att}\in R^{1\times d_{1}}$, while σ denotes the non-linear function LeakyReLU, $att_{j}$ represents the attention coefficient of the neighboring node *j*, which quantifies the importance of neighboring node *j* to the central node *o* under relation *r*. Subsequently, we use the Softmax function to normalize $att_{j}$ in order to stabilize the training process. The Softmax function transforms the attention coefficients of neighboring nodes into a probability distribution, ensuring that the sum of these weights is equal to 1:

(10) $$\begin{array}{c} \begin{array}{cc} \alpha_{j} & =softmax(att_{j})\\ & =\frac{exp(att_{j})}{\sum_{j\in N(o)}exp(att_{j})} \end{array} \end{array}$$

Then, the representation of the central node *o* is updated by calculating a weighted sum of the features from its neighboring nodes N(o) based on the normalized attention weights $\alpha_{j}$:

(11) $$\begin{array}{c} \begin{array}{cc} e_{o} & =\sigma \left(\sum_{j\in N(o)}\alpha_{j}m\left(j,r,o\right)\right) \end{array} \end{array}$$

To enhance the robustness and expressive power of DR-GAT, we apply the multi-head attention mechanism to capture the various features in multi-semantic spaces, assuming the proposed graph attention network has H attention heads and *k* stacked graph attention layers. By performing H independent attention calculations, each node will be equipped with H different node representations. To reduce the computational complexity, we average the outputs of the multi-head attention module rather than concatenating them to obtain the updated node representation ${e_{o}}^{k}$:

(12) $$\begin{array}{c} \begin{array}{cc} {e_{o}}^{k} & =\sigma \left(\frac{1}{H}\sum_{h=1}^{H}\sum_{j\in N(s)}\alpha_{j}^{h,k-1}m^{k-1}\left(s,r,j\right)\right)\\ & =\sigma \left(\frac{1}{H}\sum_{h=1}^{H}\sum_{j\in N(s)}\alpha_{j}^{h,k-1}W_{dir}^{k-1}\phi^{k-1}\left(e_{s},e_{r}\right)\right) \end{array} \end{array}$$

Finally, we define a learnable relational weight matrix $W_{r}\in R^{d_{1}\times d_{0}}$ to update the relation embeddings in the *k*-th layer:

(13) $$\begin{array}{c} \begin{array}{c} {e_{r}}^{k}={W_{r}}^{k-1}{e_{r}}^{k-1} \end{array} \end{array}$$

Through the modal-specific directed relational graph attention networks, we complete the implicit missing modal features for all entities and obtain three modal-specific embeddings of entities and relations.

#### 3.2.3. Multi-Stream Interaction Fusion

After obtaining the structural, textual, visual, and the modal-specific relational embeddings of entities, we design an interaction fusion module to facilitate the information interaction among the modal features. Conventional methods typically employ concatenation or attention mechanism to combine multi-modal information. However, these methods are not capable enough to exploit the distinctive information or capture the complementary information among various modalities. Therefore, we introduce a low-rank tensor fusion mechanism [54] that effectively aids our approach to capture both intra-modal and inter-modal information.

Firstly, we define the structural, textual, and visual features of the entity *e* as $e^{s},e^{t},e^{v}$. Subsequently, an additional element 1 is added to each modal feature, which provides a learnable bias, enhancing the ability of the proposed framework to fit single-modal information.

(14) $$\begin{array}{c} \begin{array}{c} e=\{\left(e^{s},1\right),\left(e^{t},1\right),\left(e^{v},1\right)\} \end{array} \end{array}$$

Then, a tensor cross product ⊗ is adopted to reconstruct tensor *e* into a high-dimensional tensor Z.

(15) $$\begin{array}{c} \begin{array}{cc} Z & =\overset{M}{\underset{m=1}{⨂}}e^{m}\\ & =\left[\begin{array}{c} e^{s}\\ 1 \end{array}\right]⊗\left[\begin{array}{c} e^{t}\\ 1 \end{array}\right]⊗\left[\begin{array}{c} e^{v}\\ 1 \end{array}\right]\\ & =\left(e^{s},e^{t},e^{v}\right)+\left(e^{s}⊗e^{t},e^{t}⊗e^{v},e^{v}⊗e^{s}\right)+\left(e^{s}⊗e^{t}⊗e^{v}\right) \end{array} \end{array}$$

The tensor cross product is utilized to fuse multi-modal information, where the first term represents the intra-modal of individual modal information. The second term captures the bi-modal interactions, and the last term indicates the tri-modal interactions for inter-modality fusion. Finally, the fused features can be obtained by:

(16) $$\begin{array}{c} \begin{array}{c} e=g(Z;W,b)=W\cdot Z+b \end{array} \end{array}$$

W denotes the weight matrix and $b\in R^{d_{1}}$ denotes the bias. Nevertheless, this method requires the explicit creation of a high-dimensional tensor, which leads to exponential growth in tensor dimensions as the number of modalities increases, resulting in an exponential increase in space complexity.

To address this issue, we adopt the low-rank decomposition multi-modal fusion strategy $\bar{W}$ to substitute the weight tensor W to reduce the space complexity:

(17) $$\begin{array}{c} \begin{array}{cc} \bar{W} & =\sum_{i=1}^{k}\overset{M}{\underset{m=1}{⨂}}{w_{m}}^{\left(i\right)} \end{array} \end{array}$$

*k* is a parameter utilized during the factorization, which is an artificially defined tensor rank. The factorization elements of the original weight tensor W are defined as ${w_{m}}^{\left(i\right)}$; *M* refers to the number of modalities. *m* and *i* represent the *m*-th modal and the *i*-th factorization element. The fused embedding can be formed as:

(18) $$\begin{array}{c} \begin{array}{cc} e & =(\sum_{i=1}^{k}\overset{M}{\underset{m=1}{⨂}}w_{m}^{\left(i\right)})\cdot Z+b\\ & =\sum_{i=1}^{k}\left(\overset{M}{\underset{m=1}{⨂}}w_{m}^{\left(i\right)}\cdot \overset{M}{\underset{m=1}{⨂}}e^{m}\right)+b\\ & =\Lambda_{m=1}^{M}\left[\sum_{i=1}^{k}w_{m}^{\left(i\right)}\cdot e^{m}\right]+b \end{array} \end{array}$$

$\Lambda_{m=1}^{M}$ represents the element-wise multiplication across a sequence of tensors. In our model, *M* equals to 3, and therefore the formulation can be extended as $\Lambda_{m=1}^{M}=\Lambda_{m=1}^{3}e^{m}=e^{s}\circ e^{t}\circ e^{v}$. Equation (18) can be rewritten as:

(19) $$\begin{array}{c} \begin{array}{cc} e & =(\sum_{i=1}^{k}w_{s}^{\left(i\right)}⊗w_{t}^{\left(i\right)}⊗w_{v}^{\left(i\right)})\cdot Z+b\\ & =\left(\sum_{i=1}^{k}w_{s}^{\left(i\right)}\cdot z_{s}\right)\circ \left(\sum_{i=1}^{k}w_{t}^{\left(i\right)}\cdot z_{t}\right)\circ \left(\sum_{i=1}^{k}w_{v}^{\left(i\right)}\cdot z_{v}\right)+b \end{array} \end{array}$$

Following the above equations, we can efficiently compute the fused embeddings through the modal-specific factorization elements, notably reducing the computational complexity. Moreover, due to the scalability of the low-rank decomposition method, our multi-stream framework can flexibly extend to additional modalities (e.g., numerical or acoustic features).

#### 3.2.4. Interpolation Re-Ranking Strategy

Based on the modal-specific entity embeddings, relation embeddings, and the fused features encoded by the three-stream DR-GATs, we adopt a decoder to compute the predicted scores of factual triplets and utilize the proposed interpolation re-ranking strategy to integrate the decoding scores of the three modalities. The MMKGC task is finally completed by jointly optimizing the ensemble modal-specific binary cross-entropy logits.

**Definition of decoders**: We propose two decoders to validate the effectiveness of MERGE according to ConvE [25] and InteractE [27]. ConvE is commonly used in GNN-based models for knowledge graph completion tasks and models the interactions between input entities and relations through a convolution operation. When presented with a triplet (s,r,o), ConvE transforms the embeddings of the subject entity *s* and the relation *r* into two-dimensional tensors, applying the convolution operation to compute the triplet score. The score function of ConvE for each factual triplet can be defined as:

(20) $$\begin{array}{c} \begin{array}{c} f\left(s,r,o\right)=f\left(\mathrm{vec}\left(f\left(\left[\bar{e_{s}};\bar{e_{r}}\right]*w\right)\right)W\right)e_{o} \end{array} \end{array}$$

The subsequently proposed InteractE is an approach that enhances the expressive capability of ConvE through feature permutation, checkered feature reshaping, and circular convolution. The score function of InteractE for each factual triplet can be defined as:

(21) $$\begin{array}{c} \begin{array}{c} f\left(s,r,o\right)=g\left(\mathrm{vec}\left(f\left(\phi_{chk}\left(P_{k}\right)⊛w\right)\right)W\right)e_{o} \end{array} \end{array}$$

Here, ∗ refers to convolution operation, ⊛ represents depthwise circular convolution operation, *w* denotes convolutional kernel, and [;] denotes concatenation operation. $\phi_{chk}$ represents the reshaping operation used to rearrange the embedding $e_{s}$ and $e_{r}$, so that no adjacent cells contain components from the same embedding. *f* and *g* represent the non-linear activation function ReLU and Sigmoid, respectively. The *k*-random permutations of both $e_{s}$ and $e_{r}$ are formed by $P_{k}=\left[\left({e_{s}}^{1},{e_{r}}^{1}\right);\ldots ;\left({e_{s}}^{k},{e_{r}}^{k}\right)\right]$, which is designed to capture diverse and heterogeneous interactions between entities and relations.

The scores of the factual triplets in each modality will be calculated by Equation (20) or Equation (21) with the single-modal entity embeddings $e_{s}^{m}$, relation embeddings $e_{r}^{m}$, and the fused entity embeddings $e_{o}^{fused}$. The matrix W will be used to project the head-relation embeddings to the same dimensions as the fused embeddings. We conduct comparative experiments to evaluate the decoding performance of the two decoders in Section 4.5.4.

**Re-ranking strategy**: Wang et al. [15] have observed that the best single-modal model often outperforms the joint model, which is contrary to common sense. Through a large number of experiments, they regard the original cause of such results to be overfitting. They provide solutions for this problem by optimizing the architecture of the model or employing pre-trained single-modal features to guide the training phase. Inspired by their theories, we propose a re-ranking strategy to ensemble the predicted scores achieved from each modal stream to decide the predicted entities. The ensemble of the modal ranks is not considered, as it may damage the modal-specific distribution characteristics. In fact, the distribution of the obtained scores could directly reflect the strengths and limitations of each modality for the final prediction.

**Figure 9:**
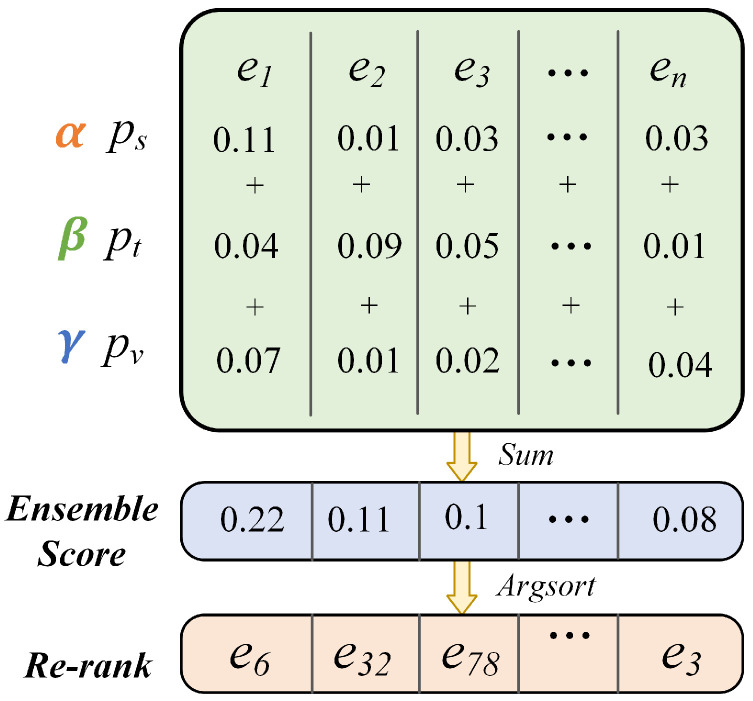
Interpolation re-ranking prediction with ensemble. $e_{1},e_{2},\ldots ,e_{n}$ denote the indices of entities. $p_{s},p_{t},p_{v}$ denote the prediction scores of structural, textual, and visual modalities. α, β, and γ are proposed to control the trade-off among three objectives.

After sorting the scores in descending order, we regard the top-ranked entity as the final prediction result. The ensemble process of our framework is depicted in Figure 9, and the ensemble prediction score can be calculated as:

(22) $$\begin{array}{c} \begin{array}{c} P=\alpha p_{stru}+\beta p_{text}+\gamma p_{visual} \end{array} \end{array}$$

$p_{s}$, $p_{t}$, and $p_{v}$ represent the prediction scores from structural, textual, and visual streams, respectively. The effect of the re-ranking strategy will be described in detail in Section 4.5.3. The hyper-parameters α∈[0,1], β∈[0,1], and γ∈[0,1] are proposed to control the trade-off among three objectives, which can be artificially adjusted. We consider the structural information as the essential information of the knowledge graph, therefore, setting α=1 to fully keep the structural predicted score. When β=γ=0, the model ignores the impact of textual and visual predicted results. β=γ=1 means an average weight is assigned to the predicted results from three modalities. By adjusting the parameters β and γ, we can adjust distinct weights to each of them. Section 4.5.5 exhibits the impact on different hyper-parameters β and γ.

**Inference and learning**: During the training phase, we utilize the binary cross-entropy with label smoothing as the loss function:

(23) $$\begin{array}{c} \begin{array}{c} L_{m}=-\frac{1}{N}\sum_{i}\left(t_{i}\cdot log\left(p_{m_{i}}\right)+\left(1-t_{i}\right)\cdot log\left(1-p_{m_{i}}\right)\right) \end{array} \end{array}$$

$t_{i}$ represents the label, taking a value of 1 for the true factual triplet, while 0 to the opposite. *m* stands for the modality, *N* forms the total number of triplets, and $p_{m_{i}}$ is the modal-specific corresponding scores to triplets. The linear combination of the logits in each modality is considered as the final training objective:

(24) $$\begin{array}{c} \begin{array}{c} L=\alpha L_{stru}+\beta L_{text}+\gamma L_{visual} \end{array} \end{array}$$

## 4. Experiment and Discussion

### 4.1. Datasets

Our model is evaluated on four publicly available datasets: FB15k-237-IMG [55] (https://github.com/mniepert/mmkb, accessed on 11 May 2018), WN18RR-IMG [34] (https://github.com/wangmengsd/RSME, accessed on 14 January 2022), DB15K-IMG [55] (https://github.com/mniepert/mmkb, accessed on 11 May 2018), and MKG-W [38] (https://github.com/quqxui/MMRNS, accessed on 18 October 2022). The textual descriptions of the first two datasets are collected by [56], while the textual descriptions of the latter two datasets are collected from DBpedia [57]. Table 3 shows the details of these datasets:

**FB15K-237-IMG**: FB15K-237 [12] is a subset of the extensive knowledge graph Freebase [2] that excludes all inverse relations to address the influence of reversible relations.

**WN18RR-IMG**: WN18 [4] is a subset originally extracted from WordNet [3], while WN18RR [25] is derived from the training set of WN18 by eliminating the inverse relations.

**DB15K-IMG**: DB15K [55] is an open-source multi-modal knowledge graph, which is a subset of DBpedia [57].

**MKG-W**: MKG-W [38] is derived from Wikidata [1]. The images of entities are extended through web search engines.

### 4.2. Evaluation Metrics

We employ three widely accepted metrics to evaluate the results of the MMKGC task. The following is a detailed description of these metrics. Here, *N* represents the total number of missing triplets, and $rank_{i}$ denotes the rank of the correct entity. For each test triplet, the model generates a ranked list of possible head/tail entities. The final result for each evaluation metric is obtained by averaging the head and tail prediction values:

• **Mean Reciprocal Rank** (MRR): MRR calculates the average of the reciprocal ranks of the correct entities across all test samples. A higher MRR indicates a stronger ability of the model to rank the correct entity toward the top.
  $$\mathrm{MRR}=\frac{1}{N}\sum_{i}^{N}\frac{1}{rank_{i}}$$
• **Mean Rank** (MR): MR is the average rank of the correct entities across all test samples. A lower value indicates a better performance.
  $$\mathrm{MR}=\frac{1}{N}\sum_{i}^{N}rank_{i}$$
• **Hits@*k***: Hits@*k* evaluates the proportion of correct answers appearing within the top-*k* candidates predicted by the model. A higher Hits@*k* value indicates a greater likelihood that the model includes the correct answer within its top-*k* predictions. In our method, *k* is set to 1, 3, and 10.
  $$Hits@k=\sum_{i}^{N}\frac{rank_{i}<k}{N},\;k=1,3,10$$

### 4.3. Implementation Details

We manually specify the hyper-parameters as follows: each modality is equipped with a one-layer graph attention network with two graph attention heads for FB15k-237-IMG, DB15K-IMG, and MKG-W and one for WN18RR-IMG. We optimize the model with Adam [58] optimizer and the learning rate is searched in {$1\times 10^{-3}$, $5\times 10^{-4}$, $1\times 10^{-4}$}. The early stopping strategy will be applied when MRR has no improvement after thirty epochs. Before feeding into the model, we project textual and visual features into the initial embedding dimension of 100, and the output embedding size of MERGE is set to 200. The best values of (β, γ) are (0.8, 0.2) for FB15k-237-IMG, (0.6, 0.2) for WN18RR-IMG, (0.3, 0.7) for DB15K-IMG, and (0.4, 0.5) for MKG-W. A Nvidia Tesla V100 GPU is adopted for acceleration.

### 4.4. Baselines

In this section, we compare our model with seven single-modal models and eight multi-modal approaches. Brief introductions to them are given as follows:

**Single-modal models**: Seven single-modal baselines are compared with our method. TransE, DisMult, ComplEx, RotatE, and InteractE are equipped with relational structure features, while CompGCN and SR-GNN are GNN-based methods:

- TransE [4]: TransE is a foundational translation-based approach that encodes entities and relations by mapping them into a continuous vector space. The object entity embedding is defined as the sum of the subject entity embedding and the relation embedding in the Euclidean space.
- DistMult [23]: DistMult employs a bilinear neural network to model multi-relational graphs, utilizing a diagonal matrix to align the representations of both entities and their relationships.
- ComplEx [24]: ComplEx uses an asymmetric scoring function to encode the relations and entities within the complex space, effectively capturing the directionality of multi-relational data.
- RotatE [20]: RotatE adopts a rotation operation to encode the relational information between nodes and relationships in the complex space. This approach leverages the geometric properties of complex numbers to capture various types of relations in knowledge graphs.
- InteractE [27]: InteractE employs feature permutation, checkered reshaping, and circular convolution to capture diverse heterogeneous interactions between entities and relationships.
- CompGCN [30]: CompGCN is a GNN-based method that incorporates multi-relational information with entities, learning the complicated interrelations between the entities in knowledge graphs.
- SR-GNN [31]: SR-GNN is a GNN-based method, which uses two semantic aggregation modules to combine the semantic similarity information between neighboring entities and the relational features of knowledge graphs.

**Multi-modal models**: Eight multi-modal baselines are compared with our method. IKRL, TransAE, RSME, CMGNN, and HRGAT are single-stream methods, while OTKGE, IMF, and MMRNS are multi-stream methods:

- IKRL [32]: IKRL utilizes the scoring function of TransE on each structural–visual entity pair for a joint prediction.
- TransAE [13]: TransAE is based on TransE by employing a multi-modal auto-encoder to integrate visual and textual information into a unified representation, regarding the fused embedding as the entity representation.
- RSME [34]: RSME is a method that proposes a filter gate and a forget gate to obtain valuable visual embeddings, applying the score function of ComplEx to model the structural information.
- OTKGE [37]: OTKGE overcomes the spatial heterogeneity of different modalities by minimizing the Wasserstein distance between the multi-modal distributions, modeling the multi-modal fusion process as a transmission process to transport different modal embeddings into a unified space.
- IMF [14]: IMF improves entity representations by designing a multi-modal fusion module to capture bilinear interactions with contrastive learning.
- HRGAT [17]: HRGAT is a GNN-based model that captures multi-source information and the graphical structure information in MMKGs.
- CMGNN [36]: CMGNN achieves multi-modal and high-order structure modeling under the graph neural networks with a contrastive learning framework.
- MMRNS^†^ [38]: MMRNS designs new negative sampling strategies for high-quality negative samples generation. We compare our method with a variant of the MMRNS model that adopts the scoring function of GC-OTE [59] for decoding, which performs best on the DB15K-IMG dataset.

### 4.5. Results and Analysis

In this section, we initially compare MERGE with various baselines on four publicly available datasets to show the efficiency of our approach. A series of ablation experiments is proposed to assess the contribution of each modality and validate the effectiveness of the modules in MERGE. The experimental analysis in the Section 4.5.7 highlights the superiority of the multi-stream framework over the single-stream framework.

#### 4.5.1. Comparison with Baselines

Experiments on the FB15k-237-IMG, WN18RR-IMG, DB15K-IMG, and MKG-W datasets have shown that the proposed MERGE outperforms both single-modal and multi-modal baselines in all evaluation metrics in the MMKGC task. The results are presented in Table 4 and Table 5. Among all presented tables in this section, the best results are marked in bold for each column.

Compared with the seven single-modal baseline methods, our approach surpasses all of them, validating the effectiveness of MERGE in utilizing multi-modal information. As a recently proposed GNN-based method, SR-GNN focuses on aggregating semantic, structural, and explicit relational information of entities. In contrast, our model not only considers the above aspects but also incorporates the integration of multi-modal information, achieving a 1.2% and 2% improvement in MRR on the FB15K-237-IMG and WN18RR-IMG datasets, respectively.

Compared with the eight multi-modal baselines, our method has demonstrated superior performance. Notably, MERGE-w/o V and MERGE-w/o T are the variants of MERGE that exclude visual and textual information, which still maintain high performance. This indicates that our method can effectively utilize the single-modal information within our framework. For the FB15K-237-IMG, WN18RR-IMG, and MKG-W datasets, MERGE-w/o V outperforms MERGE-w/o T, which is because the textual descriptions contain additional entities related to the target entity, offering broader context and deeper relational understanding. In contrast, for the DB15K-IMG dataset, MERGE-w/o T performs slightly better than MERGE-w/o V. This is due to the significant lack of textual data in DB15K-IMG, resulting in a considerable reduction in available information. In Section 4.5.7, we conduct a detailed analysis on the inferring performance of MERGE under extensive multi-modal information loss.

In the comparison of single-stream models, the results of the variant model MERGE-w/o T show that our method exhibits higher efficiency in processing and applying single-modal information compared to IKRL and RSME. As a typical GNN-based multi-modal single-stream model, HRGAT makes more use of the numerical features than MERGE. However, our method still achieves a 0.7% and 1.9% improvement in MRR on the FB15K-237-IMG and WN18RR-IMG datasets, further validating the advantage of the proposed multi-stream framework in leveraging unique features from each modality.

Compared with multi-stream models, MERGE surpasses IMF across all metrics, which justifies the validation of graph neural network in enhancing the implicit representation of missing modal information in MMKG. Both MMRNS and CMGNN adopt the contrastive learning strategy to construct positive and negative sample pairs for multi-stream encoding based on the structural information of MMKGs. In contrast, our method separately considers the structural encoding of three modalities. Compared with them, our model achieves a better experimental, demonstrating the effectiveness of modal-specific structural encoding. Although our model is slightly inferior to MMRNS in terms of Hits@10 on the MKG-W dataset, it surpasses MMRNS in other metrics, particularly achieving a 2.4% improvement in Hits@1. This highlights the superiority of MERGE in utilizing multi-modal information and making high-precision predictions.

#### 4.5.2. Modality Ablation Experiment

To verify the impact of various modalities, we conduct a series of modality ablation experiments. Table 6 shows the results of MMKGC utilizing different modalities on the FB15k-237-IMG dataset.

From rows 1–3, it is obvious to see that directly utilizing structural information obtained from the knowledge graph performs the best. This indicates that the graph structure information in MMKG can provide key relation patterns for the MMKGC task, highlighting the fundamental role of structural information. When only textual or visual features are adopted, the performance of MERGE decreases, which is due to the incomplete textual and visual corpora in MMKGs. Comparatively, the textual modality performs better than the visual modality in the MMKGC task. The possible reason may be that descriptions contain entities related to the object entity, providing a broader context and deeper relationship understanding, while images only represent the entity itself, limiting the expression of the relationship between entities.

From rows 4–6, we can observe that combining the three types of modality features in pairs can significantly improve the model’s performance, which indicates that different modal features can achieve complementary information through synergistic interactions, enhancing the understanding and predictive ability of MERGE. However, it is obvious to see that none of the combinations exceed the best model, which includes all three modalities. This demonstrates that in our approach, each modality can effectively enhance others, validating the effectiveness of MERGE in multi-modal information utilization.

#### 4.5.3. Module Ablation Experiment

To further explore the effectiveness of each component in MERGE, we design the following four variants to conduct ablation experiments on different modules within MERGE:

• **MERGE-RA**: This variant removes the attention mechanism in DR-GAT to evaluate the impact of our relational attention mechanism.
• **MERGE-RCO**: This variant eliminates the entity–relation composition operation to examine the influence of the correlations between entities and relations.
• **MERGE-RLMF**: This variant replaces the low-rank multi-modal fusion operation with the average operation to assess the impact of the fusion strategy.
• **MERGE-STRU**: This variant replaces both visual and textual embeddings with the structural embedding to evaluate the contribution of multi-modal information under the same parameter quantities.

Table 7 presents the comparison results between MERGE and its four variants on the FB15K-237-IMG dataset. From the results, it is evident to see that removing any component from MERGE leads to a performance decline.

**Impact on the Attention Mechanism**: After removing the relational attention mechanism during information aggregation, the model’s performance declines. This indicates that the attention mechanism plays a crucial role in balancing the importance of neighboring nodes, which enhances the precision of information aggregation.

**Impact on Entity–Relation Composition Operation**: When the entity–relation composition operation is removed, the model’s performance declines, which validates the significant role of entity–relation composition in enhancing relational understanding.

**Impact on Low-Rank Tensor Fusion Mechanism**: When replacing the low-rank tensor fusion operation with an average operation, the result exhibits a significant performance drop. This confirms that the low-rank tensor fusion method can effectively facilitate the inter-modal information interaction, greatly promoting the model’s understanding and the utilization of multi-modal information.

**Impact on Multi-Modal Information**: When the input embeddings of the three modal-specific directed relational graph attention networks are all replaced by randomly initialized features, the model’s performance significantly decreases. This result demonstrates that the integration of multi-modal information helps enhance the overall performance of MERGE, and the effectiveness of our model is attributed to the interaction of different modal information.

#### 4.5.4. Composition Operator and Decoder Ablation Experiment

To verify the impact of different composition operators and decoders on MERGE, we conduct ablation experiments on the FB15K-237-IMG dataset. We have defined four composition operators in Section 3.2.2 and two decoders in Section 3.2.4. The experimental results are exhibited in Table 8. It is obvious to see that different composition operators excel in optimizing different score functions.

**Analysis of ConvE Decoder**: For the ConvE decoder, the “Sub” operator outperforms the rest of the compositions. As the subtraction operation can capture the linear differences between entities and relations, it is advantageous in handling linear relationships and reducing noise from complicated relationships. The ConvE decoder is adopted to calculate interactions between entities and relations through convolution operations. Combining ConvE with the subtraction operator allows the model to extract and amplify direct linear relation features, enhancing the expression of simple relationships in MMKGs.

**Analysis of InteractE Decoder**: For the InteractE decoder, the crossover operator captures complex interactive information between entities and relations, thus is capable of capturing high-order features and non-linear relationships. The InteractE decoder is adopted to enhance the feature expressions through feature permutation, checkered feature reshaping, and circular convolution, which better captures the deeper semantic relationships between entities. Combining the crossover interaction operator with the InteractE decoder ensures efficient expression of multi-modal information, resulting in better performance in complex scenarios. Since expressing complex relationships in MMKGs is challenging, the combination of the InteractE and the crossover interaction operator performs better compared to the combination of the ConvE and the subtraction operator.

#### 4.5.5. Impact on Hyper-Parameters

In this section, we explore the effects of various hyper-parameters on MERGE. The candidate hyper-parameters are adjusted in the following ranges on the FB15K-237-IMG dataset: embedding dimensions {50, 100, 200, 300}, attention head number {1, 2, 3}, and decomposition ranks {4, 8, 16, 32, 64, 128, 256}. All experimental results are analyzed based on their Mean Reciprocal Rank (MRR). Additionally, we conduct comparative experiments on modal weights across the FB15K-237-IMG, WN18RR-IMG, DB15K-IMG, and MKG-W datasets to identify the optimal (β,γ) values for each modality.

**Figure 10:**
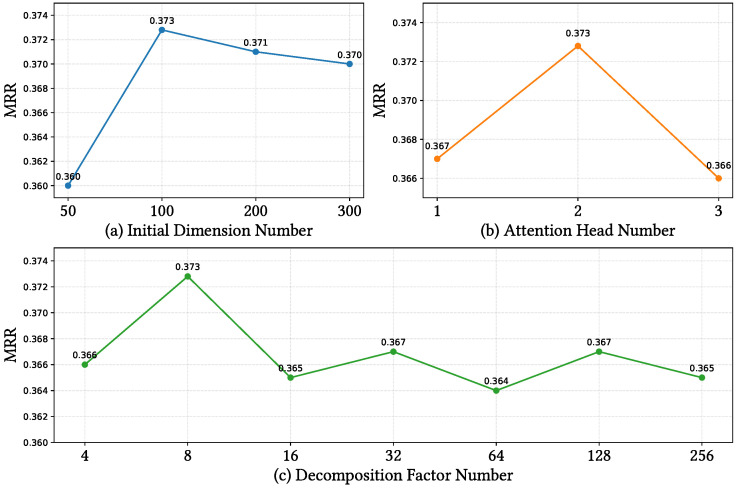
Impact of hyper-parameters on FB15k-237-IMG dataset. The vertical coordinate is the MRR metric, and the horizontal labels are embedding dimensions, attention heads. and factorization element numbers for subplots (**a**), (**b**), and (**c**), respectively.

**Impact on Embedding Dimensions**: As shown in Figure 10a, the performance of MERGE initially improves and then declines with the increase in embedding dimensions. This indicates that when the initial embedding dimension is set to 50, the model fails to sufficiently encode information from multiple modalities. After increasing the embedding dimension to 100, the model’s performance significantly improves, suggesting that a higher dimensional space can better capture the complicated relationships between multi-modal entities. However, further increasing the embedding dimensions results in a performance decline. This indicates that overly high embedding dimensions may leverage the model to memorize the training data, which hampers its generalization ability on unseen entities, resulting in the overfitting issue.

**Impact of the Attention Head Number**: Figure 10b illustrates the effect of the number of attention heads on MERGE. As the number of attention heads increases, the performance first improves and then declines. This implies that a single attention head is not sufficient to enhance the expressiveness of the model, while an excessively large number of attention heads may result in the accumulation of training errors, impacting the model’s performance.

**Impact of Decomposition Ranks**: Figure 10c shows the influence of the number of decomposition factor ranks. The results indicate that increasing the number of decomposition factors beyond a certain threshold does not improve the model performance as expected. In fact, a relatively small number of decomposition factors is sufficient to achieve good results, highlighting the importance of selecting an appropriate number for the decomposition factors to balance the model’s capacity and avoid overfitting. The eight low-rank decomposition factors are adequate to achieve interaction among multi-modal information for MERGE.

**Figure 11:**
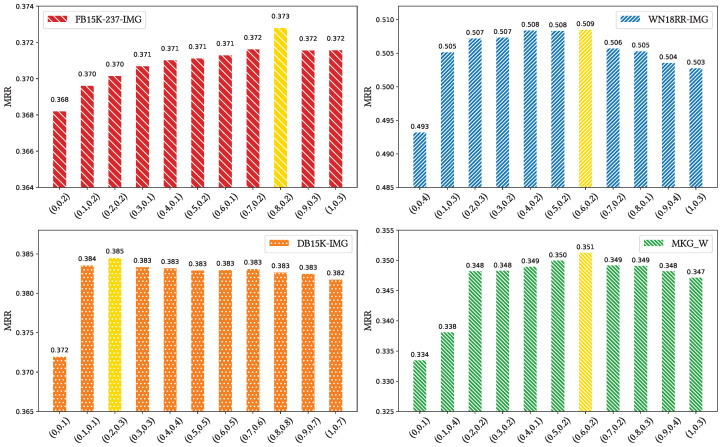
The values of (β,γ) for the FB15k-237-IMG, WN18RR-IMG, DB15K-IMG, and MKG-W datasets. The best result of each dataset is highlighted in yellow.

**Impact of (β, γ)**: To select the values of β and γ, we varied their β,γ values incrementally from 0 to 1 in steps of 0.1, respectively. The best result for each dataset is specified in yellow, which has been illustrated in Figure 11. The optimal results for (β,γ) on the FB15k-237-IMG, WN18RR-IMG, DB15K-IMG, and MKG-W datasets are (0.8, 0.2), (0.6, 0.2), (0.2, 0.3), and (0.6, 0.2), respectively. The weight for textual information is generally higher than that of the visual information, as the descriptive texts usually mention entities related to the target entity, providing more information for model to understanding the contextual relationships of entities. However, for the DB15K-IMG dataset, its textual corpora has a significant deficiency, leading to limited available information and potentially introducing more noise. Therefore, giving higher weights to the visual modality for the DB15K-IMG dataset can achieve a better performance.

#### 4.5.6. Explicit Spatial Alignment Experiment

To demonstrate the explicit spatial alignment of structural, textual, and visual modal features, we first calculate the pairwise similarity between the input and output vectors for each modality on the FB15K-237-IMG dataset. To facilitate the calculation, we reduce the dimensions of the embeddings of the three modalities by the principal components analysis (PCA) [60]. Table 9 shows the similarity calculation results for randomly selected 1024 entity node embeddings.

**Input Similarity Analysis**: The similarities between textual and visual embeddings are relatively high, which indicates that the CLIP model, trained through contrastive learning methods on text–image pairs, can effectively align the two modalities within the same semantic space.

**Output Similarity Analysis**: After being encoded by the modal-specific DR-GAT networks, the output similarities show significant improvement compared to the input similarities. This demonstrates the effectiveness of our model in achieving explicit spatial alignment for multi-modal features. In terms of the percentage increments, the textual–visual similarity shows the largest improvement, which suggests that the modal interaction processing of MERGE can further enhance the semantic relationship between the origin semantically aligned visual and textual features.

**Figure 12:**
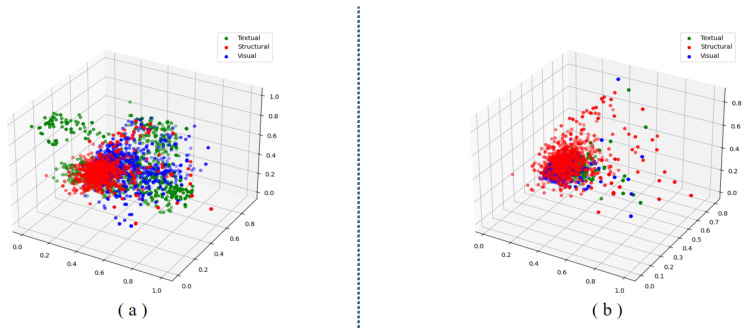
The 3-dimensional spatial distribution for the three modal features of the random 1024 entities in FB15K-237-IMG dataset. (**a**) is the spatial distribution of input embeddings, while (**b**) is the spatial distribution of output embeddings. The blue, red and green nodes stand for visual, structural, and textual modality, respectively.

**Visualization**: Figure 12 displays the 3-dimensional spatial distribution of the input and output modal-specific vectors for random selected 1024 entity nodes. It is obvious to see that compared with the distribution of input embeddings, the distribution of output embeddings becomes more clustered. This visualization indicates that our model can effectively reduce the distribution difference between initial modal features when performing graphical explicit alignment, thereby enhancing the interactions between multi-modalities.

#### 4.5.7. Analysis of Modality Information Loss on Single-Stream and Multi-Stream Architecture

In this section, we focus on exploring the differences between single-stream and multi-stream architectures in handling the information loss in multi-modal corpora. To simulate the situation of the information loss in multi-modal features, we first conduct experiments on the FB15K-237-IMG dataset when a single modality is completely missing, comparing MERGE with recent baselines, where HRGAT serves as a single-stream model, and OTKGE and IMF are multi-stream models. The experimental results are shown in Table 10.

In terms of the overall performance, the multi-stream models significantly outperform the single-stream model, highlighting the advantages of the multi-stream architecture in multi-modal information integrating. Moreover, single-stream models experience a more pronounced performance decline compared to their multi-stream counterparts, primarily due to their limited ability in leveraging the unique features of each modality. This suggests that single-stream models are less robust in managing modality information loss, as the absence of any single modality directly impairs the representation capability of the remaining ones.

Compared to other multi-stream models, our method not only achieves superior overall performance but also demonstrates relatively smaller declines in evaluation metrics when specific modalities are missing. This demonstrates the robustness of our approach in efficiently leveraging limited modality information.

Then, we define a MERGE-single framework to evaluate the inference ability of MERGE and its single-stream variant in the absence of a small amount of modal information. The MERGE-single framework first integrates structural, textual, and visual information by the low-rank tensor fusion method, then passes the fused embeddings through a single-layer DR-GAT network, and finally adopts the “InteractE” scoring function to decode the encoded triplets.

To simulate the situation of the information loss in multi-modal features, we conduct experiments on the DB15K-IMG dataset, randomly setting a certain number of textual or visual embeddings to zero vectors. Table 11 presents the MMKGC results in terms of MRR and Hits@10 for both MERGE and MERGE-single frameworks, with the number of randomly zeroed embeddings ranging {500, 1000, 1500}. δ represents the percentage decrement in MRR and Hits@10.

In terms to the overall performance, MERGE significantly outperforms its single-stream variant in both MRR and Hits@10. As the number of the missing features increases, the performance of both MERGE and MERGE-single gradually declines. Comparing the last two rows with the best result in Table 11, the result shows that with a small amount of modal information loss in a single modality, MERGE can still infer well. Specifically, when 1500 textual or visual features are initially at zero, the MRR metrics of MERGE only decrease by 1.2% and 1.0%, while those of the MERGE-single framework decrease by 4.0% and 4.7%. This verifies the robustness of our proposed multi-stream framework in addressing the incomplete multi-modal corpora.

## 5. Conclusions

Aiming at the problem of the imbalanced accumulation of multi-modal corpora in MMKGs, we innovatively propose a **M**odal **E**quilibrium **R**elational **G**raph fram**E**work (**MERGE**) to achieve implicit representation of incomplete modal information in MMKGs. This framework can efficiently complete the missing triplets in multi-modal knowledge graphs, which plays a significant role in enhancing the accuracy of knowledge inference and improving the overall understanding of complex relationships between entities across different modalities.

The proposed MERGE employs modal-specific directed relational graph attention networks for information aggregation across different modalities, employing a low-rank tensor fusion method to effectively integrate diverse modal semantic features. This ensures the alignment of varying modal features within both the explicit graphical structure and the semantic space, enhancing the interpretability of entity representation embeddings. During the inference phase, the proposed interpolation re-ranking strategy adjusts the importance of modalities while preserving the semantic integrity of each modality, ensuring high prediction accuracy even under deficient modal information. Compared with recent works, the experimental results have demonstrated the effectiveness and robustness of MERGE in the MMKGC task. Furthermore, by comparing the handling effects between the single-stream and multi-stream architectures on the MMKGC task, we find that the proposed multi-stream framework is more adept at handling large-scale incomplete multi-modal corpora in MMKGs. MERGE can accurately infer the incomplete triplets by deeply exploring the structural and semantic similarities between entities in various modalities.

Currently, MERGE still has some limitations, such as insufficient consideration of detail information in images. In future work, we will further consider adopting scene graphs for image encoding to help the model enhance its fine-grained image content understanding ability, thereby capturing the visual relationships between entities. Furthermore, the applicability of our framework may be further explored in other domains, such as healthcare, finance, and social networks, where multi-modal knowledge graphs can provide valuable insights.

## Figures and Tables

**Table 1:** The comparison between MERGE and other existing knowledge graph representation methods. “Agg.” denotes the aggregation process, “Import.” denotes the calculation of the importance scores, and “Info.” denotes the information.

Models	Entity Agg.	Relation Agg.	Neigh-Boring Import.	Multi-Modal Info.	Modal-Specific Info.
GCN [28]	✓	×	×	×	×
CompGCN [30]	✓	✓	×	×	×
SR-GNN [31]	✓	✓	✓	×	×
HRGAT [17]	✓	✓	✓	✓	×
IMF [14]	×	×	×	✓	✓
MERGE	✓	✓	✓	✓	✓

**Table 2:** The origin scoring function φ of different knowledge graph embedding models. Symbol ∘ denotes Hadamard product, ★ denotes circular convolution, σ is a non-linear activation function.

Method	Score Function
TransE	${∥e_{s}+e_{r}-e_{o}∥}_{p}$
DistMult	$\langle e_{s},e_{r},e_{o}\rangle$
HoLE	$\sigma ({e_{r}}^{T}\left(e_{s}★e_{o}\right))$
CrossE	$\sigma (e_{o}^{T}tanh\left(w_{r}\circ e_{s}+w_{r}\circ e_{s}\circ e_{r}\right))$

**Table 3:** A summary of dataset statistics.

Dataset	Entity	Relation	Train	Valid	Test	Texts	Images
FB15k-237-IMG	14,541	237	272,115	17,535	20,466	14,515	150,000
WN18RR-IMG	40,943	11	86,835	3034	3134	40,943	70,385
DB15K-IMG	12,842	279	79,222	9902	9904	9078	12,818
MKG-W	15,000	169	34,196	4276	4274	14,463	14,123

**Table 4:** MMKGC results on FB15k-237-IMG and WN18RR-IMG. The second column exhibits the used modalities in mentioned methods. “S” refers to structural information, “T” refers to textual information, “V” refers to visual information, and “N” refers to numerical information.

Method	Modality	FB15k-237-IMG				WN18RR-IMG			
		MRR	Hits@1	Hits@3	Hits@10	MRR	Hits@1	Hits@3	Hits@10
*Single-modal Methods*									
TransE [4]	S	0.279	0.198	0.376	0.441	0.243	0.043	0.441	0.532
DistMult [23]	S	0.281	0.199	0.301	0.446	0.444	0.412	0.470	0.504
ComplEx [24]	S	0.278	0.194	0.297	0.450	0.449	0.409	0.469	0.530
RotatE [20]	S	0.338	0.241	0.375	0.533	0.476	0.428	0.492	0.571
CompGCN [30]	S	0.355	0.264	0.390	0.535	0.479	0.443	0.494	0.546
InteractE [27]	S	0.354	0.263	-	0.535	0.463	0.430	-	0.528
SR-GNN [31]	S	0.361	0.269	0.399	0.546	0.490	0.456	0.504	0.556
*Multi-modal Methods*									
IKRL [32]	S + V	-	0.194	0.284	0.458	-	0.393	0.436	0.491
TransAE [13]	S + T + V	-	0.199	0.317	0.463	-	0.406	0.457	0.515
RSME [34]	S + V	-	0.242	0.344	0.467	-	0.423	0.486	0.531
OTKGE [37]	S + T + V	0.341	0.251	0.374	0.519	-	-	-	-
HRGAT [17]	S + T + V + N	0.366	0.271	0.404	0.542	0.491	0.454	0.503	0.567
IMF [14]	S + T + V	0.368	0.274	0.404	0.557	-	-	-	-
CMGNN [36]	S + T + V	0.359	0.268	0.393	0.544	0.481	0.448	0.493	0.545
**MERGE**-w/o V	S + T	0.369	**0.278**	0.402	0.555	**0.510**	0.455	0.533	**0.610**
**MERGE**-w/o T	S + V	0.360	0.266	0.396	0.548	0.487	0.449	0.503	0.557
**MERGE**	S + T + V	**0.373**	0.277	**0.410**	**0.563**	0.509	**0.457**	**0.534**	0.608

**Table 5:** MMKGC results on DB15K-IMG and MKG-W. MMRNS^†^ is a variant of the MMRNS model that adopts the scoring function of GC-OTE for decoding.

Method	Modality	DB15K-IMG				MKG-W			
		MRR	Hits@1	Hits@3	Hits@10	MRR	Hits@1	Hits@3	Hits@10
*Single-modal Methods*									
TransE [4]	S	0.249	0.128	0.315	0.471	0.292	0.211	0.332	0.442
DistMult [23]	S	0.230	0.148	0.263	0.396	0.210	0.159	0.223	0.309
ComplEx [24]	S	0.275	0.184	0.316	0.454	0.249	0.191	0.267	0.368
RotatE [20]	S	0.293	0.179	0.361	0.497	0.337	0.268	0.367	0.468
CompGCN [30]	S	0.260	0.180	0.300	0.413	0.322	0.264	0.348	0.431
*Multi-modal Methods*									
IKRL [32]	S + V	0.268	0.141	0.349	0.491	0.324	0.261	0.348	0.441
TransAE [13]	S + T + V	0.281	0.213	0.312	0.412	0.300	0.212	0.349	0.447
RSME [34]	S + V	0.298	0.242	0.321	0.403	0.292	0.234	0.320	0.404
OTKGE [37]	S + T + V	0.239	0.185	0.259	0.342	0.344	0.289	0.363	0.449
IMF [14]	S + T + V	0.323	0.242	0.360	0.482	0.345	0.288	0.366	0.454
MMRNS^†^ [38]	S + T + V	0.327	0.230	0.379	0.510	0.343	0.271	0.368	**0.470**
**MERGE**-w/o V	S + T	0.372	0.294	0.410	**0.535**	0.347	0.289	0.370	0.456
**MERGE**-w/o T	S + V	0.379	0.296	0.420	0.513	0.316	0.263	0.335	0.420
**MERGE**	S + T + V	**0.385**	**0.306**	**0.422**	0.534	**0.349**	**0.295**	**0.371**	0.451

**Table 6:** The results of modality ablation experiments on FB15k-237-IMG dataset. Stru, Text, and Visu refer to structural, textual, and visual modality, respectively. The best results are marked in bold for each column.

Stru	Text	Visu	MRR	Hits@1	Hits@3	Hits@10	MR
✓			0.363	0.272	0.397	0.545	163
	✓		0.353	0.267	0.381	0.526	142
		✓	0.330	0.244	0.359	0.498	178
✓	✓		0.369	**0.278**	0.402	0.555	146
✓		✓	0.360	0.266	0.396	0.548	178
	✓	✓	0.366	0.273	0.401	0.552	156
✓	✓	✓	**0.373**	0.277	**0.410**	**0.563**	**139**

**Table 7:** The results of module ablation experiment on FB15K-237-IMG dataset. The best results are marked in bold for each column.

Method	MRR	Hits@1	Hits@3	Hits@10	MR
MERGE-RA	0.368	0.274	0.404	0.552	**139**
MERGE-RCO	0.366	0.273	0.403	0.549	144
MERGE-RLMF	0.360	0.267	0.395	0.543	184
MERGE-STRU	0.353	0.260	0.389	0.538	243
**MERGE**	**0.373**	**0.277**	**0.410**	**0.563**	**139**

**Table 8:** The results of composition operator and decoder ablation experiment on FB15k-237-IMG dataset. The adopted composition operators includes “Sub”, “Mult”, “Corr”, and “Cross”, while the decoder types are formed as ConvE and InteractE, respectively. In this table, “→” represents reading in rows, while “↓” represents reading in columns. The best results are marked in bold for each column.

Decoder →	ConvE					InteractE				
Composition Operator ↓	MRR	Hits@1	Hits@3	Hits@10	MR	MRR	Hits@1	Hits@3	Hits@10	MR
Sub-MERGE	**0.371**	**0.275**	**0.410**	**0.560**	184	0.368	0.275	0.401	0.552	149
Mult-MERGE	0.366	0.273	0.401	0.550	156	0.366	0.273	0.402	0.551	163
Corr-MERGE	0.365	0.272	0.401	0.554	**143**	0.366	0.273	0.400	0.554	148
Cross-MERGE	0.368	**0.275**	0.406	0.554	192	**0.373**	**0.277**	**0.410**	**0.563**	**139**

**Table 9:** The pairwise similarity between the input and output vectors for each modality in FB15K-237-IMG dataset. δ is the percentage increment in similarity.

	Structural-Textual	Structural-Visual	Textual-Visual
input	0.2611	0.0682	0.2528
output	0.3108	0.1348	0.6345
δ	4.97%	6.66%	38.17%

**Table 10:** The performance between existing methods and MERGE when trained under conditions of single modality absence on the FB15k-237-IMG dataset. The best results are marked in bold for each row. The values in the subscript indicate the deviation from the best result in each column.

Text	Image	Metrics	Models			
			HRGAT	OTKGE	IMF	MERGE
		MRR	0.366	0.341	0.368	**0.373**
✓	✓	Hits@1	0.271	0.251	0.274	**0.277**
		Hits@10	0.542	0.519	0.557	**0.563**
		MRR	0.332_[−3.4]_	0.331_[−1.0]_	0.346_[−2.2]_	**0.360**_[−1.3]_
✓		Hits@1	0.231_[−4.0]_	0.239_[−1.2]_	0.261_[−1.3]_	**0.278**_[+0.1]_
		Hits@10	0.495_[−4.7]_	0.508_[−1.1]_	0.518_[−3.9]_	**0.548**_[−1.5]_
		MRR	0.355_[−1.1]_	0.335_[−0.6]_	0.353_[−1.5]_	**0.369**_[−0.4]_
	✓	Hits@1	0.256_[−1.5_	0.240_[−1.1]_	**0.268**_[−0.6]_	0.266_[−1.1]_
		Hits@10	0.527_[−1.5]_	0.506_[−1.3]_	0.549_[−0.8]_	**0.555**_[−0.8]_

**Table 11:** The inference results between MERGE and MERGE-single in the absence of a small amount of modal information on the DB15K-IMG dataset. “T-mask” and “V-mask” represent the number of embeddings that are set to zero for textual and visual features. δ represents the percentage decrease in each metric. The best results are marked in bold for each column.

Stream Architecture		MERGE				MERGE-Single			
T-Mask	V-Mask	MRR	δ	Hits@10	δ	MRR	δ	Hits@10	δ
-	**-**	**0.385**	-	**0.534**	-	**0.379**	-	**0.531**	-
500	500	0.372	1.3%	0.518	1.6%	0.362	1.7%	0.506	2.5%
1000	1000	0.358	2.7%	0.505	2.9%	0.345	3.4%	0.483	4.8%
1500	1500	0.348	3.7%	0.493	4.1%	0.330	4.9%	0.461	7.0%
1500	-	0.373	1.2%	0.521	1.3%	0.339	4.0%	0.475	5.6%
-	1500	0.375	1.0%	0.525	0.9%	0.332	4.7%	0.464	6.7%

## Data Availability

The data that support the findings of this study are openly available in github at “https://github.com/mniepert/mmkb” (accessed on 11 May 2018), “https://github.com/wangmengsd/RSME” (accessed on 14 January 2022), and “https://github.com/quqxui/MMRNS” (accessed on 18 October 2022).
